# Damage Identification in Bridges by Processing Dynamic Responses to Moving Loads: Features and Evaluation

**DOI:** 10.3390/s19030463

**Published:** 2019-01-23

**Authors:** Xiang Zhu, Maosen Cao, Wieslaw Ostachowicz, Wei Xu

**Affiliations:** 1 Department of Engineering Mechanics, Hohai University, Nanjing 210098, China; xzhu@hhu.edu.cn (X.Z.); wxu@hhu.edu.cn (W.X.); 2 Institute of Fluid-Flow Machinery, Polish Academy of Sciences, 14 Fiszera St., 80-231 Gdansk, Poland; wieslaw.ostachowicz@imp.gda.pl

**Keywords:** damage identification, dynamic response, moving load, Fourier transform, wavelet transform, Hilbert-Huang transform, heuristic interrogation, bridge

## Abstract

The detection of damage in bridges subjected to moving loads has attracted increasing attention in the field of structural health monitoring. Processing the dynamic responses induced by moving loads to characterize damage is the key to identifying damage in bridges. On this topic, various methods of processing dynamic responses to moving loads have been developed in recent decades, with respective strengths and weaknesses. These methods appear in different applications and literatures and their features have not been comprehensively surveyed to form a profile of this special area. To address this issue, this study presents a comprehensive survey of methods for identifying damage by processing dynamic responses of cracked bridges subjected to moving loads. First, methods utilizing the Fourier transform to process dynamic responses to moving loads for damage detection in bridges are examined. Second, methods using wavelet transform to process the dynamic responses to moving loads for damage characterization are examined. Third, methods of employing the Hilbert-Huang transform to process the dynamic responses to moving loads for damage identification are examined. Fourth, methods of dynamic response-driven heuristic interrogation of damage in bridges subjected to moving loads are examined. Finally, we recommend future research directions for advancing the development of damage identification relying on processing dynamic responses to moving loads. This study provides a profile of the state-of-the-art and state-of-the-use of damage identification in bridges based on dynamic responses to moving loads, with the primary aim of helping researchers find crucial points for further exploration of theories, methods, and technologies for damage detection in bridges subjected to moving loads.

## 1. Introduction

The research area of structural damage detection is important in the civil, mechanical, aerospace, military, and maritime fields [1,2,3,4]. It is generally considered that a damaged structure produces abnormal changes concealed in the structural behavior [5]. By extraction of such abnormalities, damage identification procedures can be used to evaluate the damage condition and even predict the residual service life of structures. To assess the damage condition of a structure, four operational levels are usually targeted: judging the occurrence of damage, locating damage, quantifying damage, and predicting the service life of the structure [6]. Because damage can result in variations of structural physical integrity that further cause alteration of structural dynamic characteristics, damage assessment at each level relies primarily on analyzing the changes in structural dynamic characteristics.

A bridge is a typically important structure, the importance of which has once more been stressed due to the construction of long-span bridges worldwide [7,8]. Serving in complex conditions including aging, dynamic loads, environmental influences, and overuse, a bridge suffers various types of damage, such as cracking and fatigue. Compared with environmental factors like corrosion, material loss, and support deterioration, a moving load is a more crucial feature that affects bridge condition [9]. A moving load produces greater stresses and deflections than the equivalent static load. In the interests of safety and normal operation, damage identification in bridges is significant in structural damage detection research. In particular, damage detection in bridges subjected to moving loads is an active research focus.

The equation of motion for a bridge bearing a crack is the foundation of development of damage identification methods. Such an equation is usually created by introducing a term for crack representation into the equation of motion of an intact bridge. For model analysis, a moving load is usually applied as a massless force, a mass, an oscillator, an inertial force, or a modeled vehicle, and a bridge is modeled by an Euler-Bernoulli beam.

With these conditions, the equation of motion for an intact bridge subjected to a moving load *M* with velocity *ν*(*t*) can be expressed as [10]:

(1) $$EI\frac{\partial^{4}y}{\partial x^{4}}(x,t)+m\frac{\partial^{2}y}{\partial t^{2}}(x,t)=-M\left[{g+v}^{2}\frac{\partial^{2}y}{\partial x^{2}}+2v\frac{\partial^{2}y}{\partial x\partial t}+\frac{\partial^{2}y}{\partial t^{2}}\right]\delta (x-\hat{x}(t)),$$

where *y*(*x,t*) denotes the vertical displacement of the beam at the position *x* and time *t*, *m* is the mass per unit length, *E* is the Young’s modulus, and *I* is the area moment of inertia; $\hat{x}(t)\;=\;vt$ signifies the instantaneous load position along the beam with the velocity v; δ(x) implies the Dirac delta function; *g* denotes the gravitational acceleration; *L* refers to the beam length. With this model, many researchers have studied structural dynamic response problems induced by moving loads in intact bridge–vehicle systems [11,12,13].

When a crack is introduced in the bridge, it can be modeled by a rotational spring with sectional flexibility. The compatibility conditions at the position of the crack for displacement, slope, bending moment, and shear force are represented as [14]:

(2) $$\begin{array}{l} y_{\left(1\right)}(x_{1}^{-},t)=y_{\left(2\right)}(x_{1}^{+},t),\\ {y\prime }_{\left(2\right)}(x_{1}^{+},t)-{y\prime }_{\left(1\right)}(x_{1}^{-},t)=\theta {y\prime }_{\left(2\right)}(x_{1}^{+},t),\\ {y\prime \prime }_{\left(1\right)}(x_{1}^{-},t)={y\prime \prime }_{\left(2\right)}(x_{1}^{+},t),\\ {y\prime \prime \prime }_{\left(1\right)}(x_{1}^{-},t)={y\prime \prime \prime }_{\left(2\right)}(x_{1}^{+},t), \end{array}$$

where $x_{1}$ denotes the location of the crack, θ signifies the non-dimensional sectional flexibility of a crack, which represents the crack extent, the symbols $x_{1}^{-}$ and $x_{1}^{+}$ denote the positions immediately above and below the crack position $x_{1}$, $y_{\left(1\right)}$ and $y_{\left(2\right)}$ denote the vertical displacement of the beam at position $x_{1}^{-}$ and $x_{1}^{+}$, and the sub-index in the parenthesis represents the segments (sub-beams) of the system.

In a bridge involving a crack, the crack divides the bridge into two segments with the equation of motion for each segment described by Equation (1). The simultaneous equations of motion for two segments, compatibility conditions in Equation (2), and the boundary conditions of the whole bridge can derive the dynamic response *y*(*x,t*) of the bridge with a crack. It can be found in [15] for solving the dynamic response of a cracked bridge.

Various studies have demonstrated that the dynamic characteristics of bridges bearing cracks due to moving loads convey sufficient information about damage [14,16]. The dynamic characteristics used to portray damage may involve deflection [17,18,19,20,21,22,23], mode shape [24,25,26], acceleration [27,28,29], moving principal component analysis [30,31], energy ratio of the vibrational response [32], virtual distortion [33], dynamic response sensitivity [34], footprints of the dynamic amplification factor [35], differences in modal curvature [36], and strain sensing [37]. It is worth mentioning that Majumder and Manohar [38] presented a time domain approach for utilizing vibration data induced by a moving vehicle to assess damage in bridge structures based on the framework of finite element modeling.

This study provides deep exploration and comprehensive examination of methods for identifying damage in bridges from the perspective of processing dynamic responses to moving loads. The tools of signal processing are related to the Fourier transform (FT) [39,40,41,42,43,44,45,46,47,48,49,50,51,52,53], wavelet transform (WT) [54,55,56,57,58,59,60,61,62,63,64,65,66,67,68,69,70,71,72,73,74,75,76,77,78,79,80,81,82,83,84,85,86,87,88,89,90,91,92,93,94,95,96,97,98,99], Hilbert-Huang transform (HHT) [100,101,102,103,104,105,106,107,108,109,110,111,112,113,114,115,116,117], and heuristic interrogation of damage (HID) [118,119,120,121,122,123,124,125,126,127,128,129,130,131,132,133,134,135,136,137,138,139,140,141,142]. The rest of this study is divided as follows: Section 2 examines methods utilizing the FT to process dynamic responses to moving loads for damage detection in bridges. Section 3 examines methods using WT to process the dynamic responses to moving loads for damage characterization in bridges. Section 4 examines methods of employing the HHT to process the dynamic responses to moving loads for damage identification in bridges. Section 5 examines methods of dynamic response-driven heuristic damage interrogation in bridges subjected to moving loads. The last section presents future research directions for advancing the development of damage identification relying on processing dynamic responses to moving loads.

## 2. FT-Processed Dynamic Responses to Moving Loads for Damage Detection

The FT is a linear integral transform that decomposes a signal (function) from the time domain to the frequency domain [39,40], revealing the frequency components of the signal. For damage identification in bridges, the dynamic responses to moving loads, e.g., acceleration, velocity, displacement, can be easily acquired from a bridge using advanced sensing devices. In most cases, however, the temporal characteristics of dynamic responses cannot reflect the behavior and condition of a bridge in a direct way. To this end, the FT is most frequently used to convert temporal dynamic responses from the time domain to the frequency domain, deriving dynamic quantities such as natural frequency and transmissibility in the frequency domain. Each of these alternative dynamic quantities arising from responses to moving loads provides the possibility of detecting damage in bridges.

(1) Natural frequencies

Natural frequency is an intuitive derivative of dynamic responses of bridges subjected to moving loads [41]. In damage detection, natural frequency can be obtained using the fast FT (FFT) to deal with the measured dynamic responses of bridges subjected to moving loads [42]. The dynamic responses of bridges can be measured easily using sensing technologies such as conventional wire sensors for velocity and acceleration and modern wireless sensors with the advantages of wireless transmission of data, lower cost, and no power requirement during measuring operations. The principle of reflecting damage using natural frequencies can be illustrated on a girder of a bridge. The natural frequency of a simple girder can be expressed as [43]:

(3) $$f=\frac{\pi }{2L^{2}}\sqrt{\frac{EIg}{W}},$$

where *f* represents the natural frequency; *g* refers to the gravitational acceleration; *W* stands for the bridge weight per unit length; *L* denotes the beam length, *E* is the Young’s modulus; *I* is the second moment of the area. Damage causes alteration in *E* and/or *I*, in turn inducing change in *f*. Therefore, a change in *f*, recognized by processing the dynamic responses of a bridge subjected to moving loads, can manifest damage in the bridge under investigation.

Yang et al. [44] used the FFT to extract natural frequencies from the dynamic responses of cracked inhomogeneous Euler-Bernoulli beams subjected to a concentrated moving load along with an axial force. The natural frequencies obtained were employed to identify damage in the beams. The results showed that crack could cause natural frequencies to decrease; moreover, the location of the crack influenced the degree of decrease in natural frequencies.

It should be noted that, despite the applicability reported, the use of natural frequency to characterize damage still fails to address the general acknowledgment that natural frequency is not sensitive to slight damage in bridges.

(2) Spectrogram

Spectrograms have been presented for analyzing the dynamic responses of a multiply cracked beam subjected to a general moving load. A spectrogram is a visual representation of the spectrum of frequencies in a time signal. A common spectrogram format is a graph with geometric axes; the horizontal axis represents time and the vertical axis represents frequency. Once acceleration of a vehicle is obtained from sensors, the spectrogram of the signal is obtained and analyzed to check the appearance of the bridge’s fundamental frequency. The spectrogram ($\Phi_{X}$) of a signal X(t) in the time and frequency axes can be mathematically expressed as [45]:

(4) $$\Phi_{X}(t,\omega )={\left|X^{*}(t,\omega )\right|}^{2},$$

where X* denotes the short-time FT of the signal. It is defined as [45]:

(5) $$X^{*}(\tau ,\omega )=\int_{-\infty }^{+\infty }X(t)W(t-\tau )e^{-j\omega t}dt,$$

where *X*(*t*) is the signal to be transformed; *W*(*t*) is the window function.

Lalthlamuana and Talukdar [45] performed spectrographic analyses of vehicle acceleration responses for theoretical study of the visibility of a bridge’s natural frequencies. They analyzed the responses of the moving load vertical acceleration to search for the bridge natural frequency. In the study, the mass ratio 0.081 of the bridge–vehicle provided optimal resolution to detect the fundamental frequency of the bridge from vehicle acceleration. This approach seems promising and cost effective because instrumentation of a vehicle is easier than that of a bridge.

In a different approach, Keenahan et al. [46] used the spectra of the processed acceleration responses of a moving load to identify changes of the bridge’s damping. Via prior knowledge of the first natural frequency of the bridge, it was possible to locate and differentiate within the acceleration spectra and compare recorded frequencies to the tabulated values. The spectra of the acceleration responses were obtained at all the simulated damping levels. For higher levels of damping, the magnitude of the peak decreased. Hence, a change in damping resulting from bridge damage could be detected. It was possible to identify bridge frequency and changes in damping for a quarter-car on a smooth road profile, but it is more difficult on a rough road profile. However, poor condition of a bridge deck surface prevents the bridge fundamental frequency from appearing with adequate clarity in the spectrogram.

(3) Power spectral density

Dynamic responses of bridges subjected to moving loads can be acquired from the acceleration responses of the moving load. To obtain natural frequencies and mode shapes of the bridge, the power spectral density matrix of the acceleration response in each frequency is decomposed by taking the singular value decomposition of the matrix as [47]:

(6) $${\hat{G}}_{yy}(j\omega_{i})=U_{i}S_{i}U_{i}^{H},$$

where ${\hat{G}}_{yy}(j\omega_{i})$ denotes the power spectral density matrix of the acceleration responses of the moving load on the bridge; the matrix $U_{i}=\left[u_{i1},u_{i2},\ldots ,u_{im}\right]$ refers to a unitary matrix, in which $u_{ij}$ denote the singular vectors; $S_{i}$ is a diagonal matrix in which $s_{ij}$ are the scalar singular values. If the singular values acquired from the acceleration responses of the moving load are plotted, dominant peaks indicate the natural frequencies of the bridge and corresponding singular vectors are the mode shapes of the structure. This is an output-only modal analysis method in the frequency domain called frequency domain decomposition (FDD), which was first proposed by Brincker et al. in [48].

Based on the FDD above, Malekjafarian and O’Brien used finite element numerical simulation to obtain the acceleration responses of a moving vehicle [47]. They conducted three simulations for bridge health monitoring based on single vehicle measurement, multiple vehicle measurements, and measurements from two connected vehicles. As well, random excitation was applied to the bridge in order to improve the bridge frequency at vehicle acceleration.

The most important advantage of the FDD method is that only the response signals are measured and the test structure is excited by the ambient excitation [49]. The FDD method provides a smooth diagram for identifying both bridge and vehicle frequencies, in which it is more effective than the FFT. In the third spectral method, the use of measured responses from multiple different vehicles can lead to more efficient results for comparisons with different random excitation of the bridge. The FDD method could be an efficient alternative to the FFT for bridge health monitoring using the response to an instrumented vehicle.

(4) Transmissibility function

Transmissibility function (TF) is a typical frequency domain dynamic quantity derived from the dynamic responses of bridges subjected to moving loads [50]. The concept of TF originates from the definition of the frequency response function (FRF) [51,52]. When subjected to an excitation at point $x_{m}$, given by a moving load, a bridge and the moving load generate structural responses such as displacement, velocity or acceleration, denoted by a(t) and b(t). The FT is used to process a(t) and b(t) individually, leading to their respective forms in the frequency domain: A(ω) and B(ω). The FRF is defined as the ratio between the bridge response and the moving load response [53]:

(7) $$H_{m}(\omega )=\frac{A(\omega )}{B(\omega )},$$

and the TF is defined as the ratio of this pair of FRFs [53]:

(8) $${TF}_{mn}(\omega )=\frac{H_{m}(\omega )}{H_{n}(\omega )},$$

where ω is angular frequency and m, n are the numbers of node locations. Equation (8) shows that the TF can be calculated directly from the FT of the bridge response (output) and the moving load response (input).

Kong et al. [53] used a running vehicle to act as excitation of moving loads and measured the displacement of the vehicle. Then the output bridge responses of displacement at points m and n were measured. The TF between the FRF of the bridge response and the vehicle response at various locations could be expressed as [53]:

(9) $$TF_{mn}=\frac{\sum_{j=1}^{\infty }\phi_{j}(x_{m})\phi_{j}(d)H_{j}(\omega )}{\sum_{j=1}^{\infty }\phi_{j}(x_{n})\phi_{j}(d)H_{j}(\omega )},$$

where $x_{m}$, $x_{n}$ denote node locations on the bridge; $\phi_{j}(x)=\sqrt{2}\sin(j\pi x/L)$ is the sinusoidal form of the mode shapes for a simply supported beam; $H_{j}(\omega )=1/\left[(\omega_{j}^{2}-\omega^{2})+{i\beta }_{j}\omega \right]$, $\omega_{j}={\left(j\pi /L\right)}^{2}\sqrt{EI/M_{b}}$, where L refers to the length of the bridge; $M_{b}$ is unit mass; and EI is the flexible stiffness of the bridge.

Using the TF, the transmissibility damage indicator (TDI) was developed to distinguish the differences in transmissibility between the intact and damaged bridges, given by [53]:

(10) $$\mathrm{TDI}=\frac{1}{N_{\omega }}\sum_{\omega =\omega_{1}}^{\omega_{2}}\frac{{\left|\sum_{j=1}^{M}\sum_{n=1}^{N}\sum_{m=1}^{N}{}^{d}{\mathrm{TR}}_{mn}^{\left(j\right)}(\omega )\cdot {\mathrm{TR}}_{mn}^{\left(j\right)}(\omega )\right|}^{2}}{\sum_{j=1}^{M}\sum_{n=1}^{N}\sum_{m=1}^{N}{\mathrm{TR}}_{mn}^{\left(j\right)}\cdot {\mathrm{TR}}_{mn}^{\left(j\right)}\cdot \sum_{j=1}^{M}\sum_{n=1}^{N}\sum_{m=1}^{N}{}^{d}{\mathrm{TR}}_{mn}^{\left(j\right)}\cdot {}^{d}{\mathrm{TR}}_{mn}^{\left(j\right)}},$$

where M, N, $N_{\omega }$ denote the number of applied forces, the number of measured positions, and the number of frequencies respectively; ${\mathrm{TR}}_{mn}^{\left(j\right)}$ refers to the transmissibility between positions m and n under the applied force at position j; ${}^{d}{\mathrm{TR}}_{mn}^{\left(j\right)}$ denotes the transmissibility between positions m and n under the applied force at position j for the damaged case. The important TDI features are: (1) usefulness for damage detection; (2) vehicle velocity having a slight impact on the TDI of the bridge response but a great influence on the TDI of the vehicle response, especially in cases of small damage; (3) as excitations, random traffic flow is much preferable to regular traffic flow, and can be applied to actual bridges.

When the TF is used to reflect damage, it exhibits three significant characteristics: (1) it is sensitive to slight damage; (2) it avoids measurement of the excitation; and (3) it can largely suppress measurement noise.

Nevertheless, several difficulties for frequency domain methods exist [21]: (1) the necessary baseline of previously measured data of the intact structure is often unavailable; (2) frequency, as a global modal parameter, is insensitive to local damage; (3) existing sensor measurements of frequency are not accurate enough; (4) frequency can be influenced by environmental conditions such as temperature or humidity. Thus it is difficult for data-processing techniques to deal with transient signals and nonlinear systems, a drawback that has prevented practical application of available vibration-based techniques [115]. Despite the disadvantages, traditional Fourier spectral techniques can decompose the signal of harmonic components into frequencies. The frequencies are averaged over the time domain and restricted to linear systems and stationary processes. Generally, modal frequency methods can only define large damage and detect damage-induced natural frequency modifications. They lack the facility to determine the location of damage. However, the techniques perform poorly in most applications because modal frequencies are not very sensitive to incipient structural damage, cannot provide sufficient useful information regarding damage, and are easily affected by environmental conditions. More sophisticated modal techniques, based on changes in curvature mode shapes, are more sensitive and can locate damage under ideal conditions, but they are strongly influenced by ambient noise and numerical errors. Though vibrational mode shapes involve more damage information, the measurements are often incomplete and can cause more errors. In summary, the use of FT methods based on modal properties has disadvantages for crack identification. Hence, researchers have developed more efficient methods to solve existing problems. Signal processing methods such as WT, HHT, and HID methods, are widely used at this time.

## 3. WT-Processed Dynamic Responses to Moving Loads for Damage Characterization

The WT is a mathematical tool that can decompose a temporal signal into a summation of time domain basis functions for measuring the time-frequency variations of spectral components [54,66]. This simultaneous time-frequency decomposition gives the WT a special advantage over the traditional FT in nonstationary signal analysis. Unlike the FT, in which sinusoidal functions are always used as the basis of decomposition, other basis functions can be selected for wavelet shapes according to signal features, providing a powerful tool for portraying local features of signals. The WT has been applied in the field of structural damage detection for predicting the occurrence of damage [55,56,57,58], locating damage [59,60,61], and quantifying damage [62,63,64,65]. In particular, it has been increasingly used in the detection of damage subjected to moving loads [73,74,75,76,77,78,79,80,81,82,83,86,87].

The WT is defined as follows [66]. A wavelet *ψ* is a function of zero average:

(11) $$\int_{-\infty }^{+\infty }\psi (t)dt=0.$$

The basis function is defined by two parameters: scale and translation in wavelet analysis. ψ is dilated with a scale parameter s and translated by u:

(12) $$\psi_{u,s}(t)=\frac{1}{\sqrt{s}}\psi (\frac{t-u}{s}).$$

The WT of f at scale s and position u is computed by correlating f with a wavelet atom:

(13) $$Wf(u,s)=\int_{-\infty }^{+\infty }f(t)\frac{1}{\sqrt{s}}\psi^{*}(\frac{t-u}{s})dt.$$

The term “compact support” is frequently mentioned in the definition of a basis function. It means that the values of the basis function are non-zero for finite intervals. This property enables efficient representation of signals with localized features. Pakrashi et al. [67] presented experimental monitoring of the evolution of an open crack in a beam by using a wavelet coefficient to process the beam-vehicle interaction signal. Their study was particularly useful in the context of continuous on-line bridge health monitoring. Previous research studies using only one wavelet scale were effective in detecting large cracks, but the detection of small levels of damage was unreliable. To overcome this limitation, Hester and González [68] developed a novel wavelet-based approach utilizing a range of scales, which was more sensitive to damage than using just a single scale to detect damage in a bridge subjected to a moving load. The method utilized the acceleration signal in place of the deflection signal to calculate the wavelet energy content in a bridge based on a vehicle-bridge finite element interaction model. However, the acceleration signal introduced two problems to the approach: (1) the vehicle axles on the bridge led to sharp singularities in the acceleration signal; and (2) the existence of the road profile made the acceleration signal rougher than that caused by a moving constant load model. Discussion of the method illustrated that a crack could only be identified under a series of limited conditions based on wavelet energy content.

### 3.1. CWT-Based Methods

#### 3.1.1. Definition of the CWT

One method based on the WT is the continuous wavelet transform (CWT). A square-integrable signal f(x) of the CWT method is defined as [69]:

(14) $$W(a,b)=f(x)⊗\psi_{b}(x)=\frac{1}{\sqrt{b}}\int_{-\infty }^{+\infty }f(x)\;\psi^{*}\left(\frac{x-a}{b}\right)\;dx,$$

where x is time or space; ⊗ denotes the convolution of two functions; $\psi_{b}(x)$ refers to the dilation of ψ(x); a, b denote the translation indicating the locality and the scale factor, respectively; $\psi^{*}\left(x\right)$ is the complex conjugate of ψ(x), which is a mother wavelet satisfying the following admissibility condition: $\int_{-\infty }^{+\infty }\frac{{\left|\Psi (\omega )\right|}^{2}}{\left|\omega \right|}d\omega <+\infty$, where Ψ(x) refers to the FT of ψ(x). The inclusion of the integral in the equation requires that ψ(0)=0, i.e.,$\int_{-\infty }^{+\infty }\psi (x)dx=0$, and then Equation (14) can be expressed as [69]:

(15) $$W(a,b)=\langle f(x),\psi_{a,b}^{*}(x)\rangle .$$

Hence, CWT is a collection of inner products of a signal f(x) and the translated and dilated wavelets $\psi_{a,b}(x)$ [70,71,72].

#### 3.1.2. CWT for Damage Detection Subjected to Moving Loads

Many researchers have conducted significant studies regarding damage detection in bridges subjected to moving loads using the CWT. For the acceleration responses of a bridge, Vaidya and Chatterjee [73] employed the CWT to analyze the acceleration responses of a cracked beam for damage location and quantification. Use of the CWT could lead to the discovery of the discontinuity contained in the acceleration signal caused by the crack. The wavelet coefficient peak occurs at the position of discontinuity, indicating the presence and location of the crack. The value of the wavelet coefficient peak could assist in identifying the extent of the crack. The proposed method has shown the ability to detect damage even for higher velocities of moving loads and small cracks in the beam. Manuel et al. [74] employed the CWT technique to analyze the vertical acceleration responses of a beam subjected to moving loads to locate and quantify the damage. The CWT was used to calculate the wavelet energy from the CWT coefficients along the selected range of scales in which a significant peak indicated the presence of damage, whereas the position and magnitude of the maximum peak of the wavelet energy revealed the location and extent of the damage. Precision in crack location was increased by filtering the signals, removing the border effects of the CWT and calculating the average of wavelet energies equivalent to different measuring points. Numerical and experimental results of the method, with good agreement, demonstrated that damage could be identified and localized even with a large amount of noise. In a similar study, Manuel et al. [75] utilized two different models, an analytical model and a finite element model, to analyze the acceleration and displacement responses of the beam subjected to moving loads.

For deflection or displacement responses of a bridge, González and Hester [76] used the wavelet technique to analyze the midspan deflection response of a beam subjected to a calibrated vehicle for damage detection. Their results showed that the use of multiple measurement points to obtain dynamic responses more accurately identified smaller cracks than the use of just one measurement location and that wavelets could be employed to process the response of a structure subjected to a moving load for damage location. Zhu and Law [77] applied the CWT to analyze the dynamic deflection responses of a cracked beam subjected to a moving load for crack detection. The CWT of measured displacement $w(x_{m},t)$, adopting the Mexican Hat wavelet as the multiscale differential operator of the wavelet, was represented as [77]:

(16) $$Ww(\hat{x}(t),s)\;=\;w(x_{m},t)⊗\psi_{s}(\hat{x}(t))\;=\;s^{2}\frac{d^{2}}{d\hat{x}{\left(t\right)}^{2}}(w(x_{m},t)⊗\theta_{s})(\hat{x}(t)),$$

where ⊗ refers to the convolution of two functions; $w(x_{m},t)$ denotes the displacement function of the beam; $\hat{x}(t)$ implies the position of the moving load at time t; $\theta_{s}$ signifies the Gaussian function. Based on the CWT, the method located cracks from sudden changes in the spatial variation of the transform responses and used a damage index that connected crack size to the coefficients of the WT to estimate the relative extent of cracks. Numerical simulation and experimental verification of the method validated that multiple instances of damage could be located accurately with the impact of measurement noise, speed and magnitude of the moving load, and measurement location. Yu et al. [78] used CWT and the Lipschitz exponent to analyze the deflection of a bridge subjected to a moving load for damage localization and quantification. Moreover, the influences of multiple damage, different degrees of damage, load magnitude, load velocity, and different sensor locations were discussed. The authors concluded that: (1) the CWT of the response of the bridge subjected to a moving load could be employed to identify multiple damage, requiring no information from the undamaged bridge; (2) a higher degree of damage could result in a smaller Lipschitz exponent; (3) damage was easier to identify when a sensor was located near the damage; and (4) the identification result could be influenced by the load velocity, and high velocity especially could significantly affect the precision of that identification. An et al. [79] applied CWT to analyze the displacement time histories of a moving spring-mass for damage detection on a bridge beam. The displacement response of the moving spring-mass unit was obtained by modal decomposition theory; the natural frequencies and mode shapes of the cracked beam were resolved using the transfer matrix method. Numerical simulation results showed that: (1) the crack location was identified by the position of the maximum value in the wavelet coefficient curve, requiring no baseline; (2) the extent of damage could be detected using a reference database of the damage index based on the wavelet coefficient; (3) the identified position of a crack in the beam could be unaffected by changes in parameters such as the velocity of the moving load, stiffness, and damping; (4) the frequencies of the beam displayed obvious changes when a crack occurred at the high absolute value of a mode shape, whereas the changes in frequencies remained stable at the stationary point of a mode shape; and (5) the frequency changes were insensitive to small cracks and low-order frequencies were more sensitive than higher-order ones. Khorram et al. [80] adopted the CWT technique combined with factorial design to process the deflection responses of a beam subjected to a moving load for multiple crack detection. The value of the CWT coefficient at the points of cracks was assumed as a damage index (DI), the maximum of which showed the locations of cracks. Applying factorial design, the DI could be used to detect the extent of each crack independently. Using a novel technique for defining the velocity of the moving load and normalizing the deflection, the DI was independent of the beam’s material properties. Based on the DI, a novel multivariable curve-fitting approach that did not depend on the responses of the intact beam could independently detect crack extent of more than 5% depth of the beam’s height with a noise level up to 10% in a multiply cracked beam. On the basis of their method, Khorram et al. [81] compared two wavelet-based damage detection approaches to find the location and size of a crack in a beam subjected to a moving load. The first approach, designated the ‘fixed sensor approach’, located a sensor at midspan of the beam using the CWT coefficient of the time-varying deflection attributed to the beam. The second approach analyzed the CWT coefficient of a sensor attached to the moving load. Notably, the approach with a moving sensor outperformed the fixed sensor approach.

For the velocity response of a bridge, Zhang et al. [82] utilized the CWT to locate cracks based on the bridge subjected to a moving load. The CWT, which could provide information in time and scale simultaneously, was used to find the singularity point on the velocity response at which a dip in the wavelet coefficients suggested the damage location. Based on the velocity response analyzed by the WT, numerical studies have illustrated that: (1) the damage location can be identified by the WT with a single scale; (2) the value of the wavelet coefficients can be utilized to evaluate the damage intensity; (3) different damage locations cause different deflections of the beam; (4) damage can be located with a noise level up to 6%; and (5) the proposed damage detection method is suitable for loads moving at relatively slow velocity but not at fast speeds.

For the displacement of a moving load, Nguyen and Tran [83] employed the CWT and inverse WT to analyze the dynamic response of a beam-like bridge subjected to a moving vehicle. The dynamic response of the bridge–vehicle system was directly obtained from the displacement of the moving vehicle that caused small distortions at crack locations when it moved on the bridge. Those small distortions could be effectively detected by the WT technique, in which the positions of large values (peaks) indicated crack locations. The inverse WT in the method was rewritten as [83]:

(17) $$f(t)\;=\;C_{g}^{-1}\int_{-\infty }^{+\infty }a^{-2}[\int_{-\infty }^{+\infty }W(a,b)\psi_{a,b}db]da,$$

where $C_{g}=2\pi \int_{-\infty }^{\infty }\frac{{\left|\overset{\wedge }{\psi }(\xi )\right|}^{2}}{\left|\xi \right|}d\xi <\infty$. Numerical simulation results of the approach revealed that the method was applicable for low-velocity movements and small cracks up to 10% depth of the beam height and noise level up to 6%, but was not recommended for high velocity movements.

### 3.2. DWT-Based Methods

#### 3.2.1. Definition of the DWT

The discrete wavelet transform (DWT) represents signals by approximations and details. Finally, the signal f(t) is expressed as [84]:

(18) $$f(t)=A_{J}+\sum_{j\leq J}\sum_{k\in Z}a_{j,k}\psi_{j,k}(t),$$

where Z denotes the set of positive integers and j,k∈Z; $\psi_{j,k}(t)=2^{j/2}\psi (2^{j}t-k)$; $A_{J}=\sum_{j>J}\sum_{k\in Z}a_{j,k}\psi_{j,k}(t)$; $a_{j,k}=\int_{-\infty }^{+\infty }f(t)\psi_{j,k}^{*}(t)dt$. The DWT is computationally efficient because it adopts dyadic scales and translations, whereas the CWT requires considerable calculation effort to find the coefficients at each individual value of the scale parameter. The CWT uses only a wavelet function, whereas the DWT uses a scaling function in addition to the wavelet function. Not all wavelet functions have scaling functions; only orthogonal wavelets have their own scaling functions. The DWT is comparatively beneficial for on-line structural health monitoring because it can efficiently identify the time and frequency changes caused by stiffness degradation.

#### 3.2.2. DWT for Damage Detection Subjected to Moving Loads

To obtain the acceleration response of a bridge, Li and Law [85] employed a dynamic acceleration response reconstruction-based approach to track damage in a target substructure subjected to moving vehicular loads. The method formulates the relationship between two sets of time domain response vectors from the target substructure subjected to moving loads, with the transmissibility matrix based on the impulse response function in terms of the DWT in the wavelet domain. It uses the finite element model of the intact target substructure and the measured dynamic acceleration responses from the damaged target substructure to identify damage, without needing the time histories or information about moving loads. The acceleration response ${\ddot{x}}_{q}(t_{n})$ at sensor location q and time instant $t_{n}$ taken from the structure subjected to a moving load $P_{\mathrm{int}}(t)$ can be obtained as [85]:

(19) $${\ddot{x}}_{q}(t_{n})=\int_{0}^{t_{n}}{\ddot{h}}_{q,l_{\tau }}(t_{n}-\tau )P_{\mathrm{int}}(\tau )d\tau ,$$

in which ${\ddot{h}}_{q,l_{\tau }}(t)$ denotes the unit impulse response function of the structure; $l_{\tau }$ is location of the moving load. The input–output relationship of the structure subjected to moving loads can also be expressed as [85]:

(20) $${\ddot{x}}_{q}={\ddot{h}}_{q,\mathrm{int}}^{DWT}P_{\mathrm{int}}^{DWT},$$

where ${\ddot{h}}_{q,\mathrm{int}}^{DWT}$ and $P_{\mathrm{int}}^{DWT}$ signify the DWTs of ${\ddot{h}}_{q,l_{\tau }}(t_{n}-\tau )$ and $P_{\mathrm{int}}(\tau )$. The proposed method effectively assessed simulated local damage corresponding to 5% noise in the measured data using the adaptive Tikhonov regularization technique. Nevertheless, numerical results showed that the accuracy of response reconstruction and the subsequent damage identification would be affected by sensor selection.

For the displacement response of a bridge, He and Zhu [86] used the DWT to analyze the dynamic displacement response of a simply supported beam with local stiffness reduction subjected to a moving load. The dynamic displacement response of the beam is a superposition of two components, namely the moving-frequency component corresponding to the moving load and the natural frequency component of the beam. The moving-frequency component is preferred to the other component in damage localization examined by the closed-form solution, which is derived based on the modal perturbation and modal superposition method. Then, the moving-frequency component is separated from the total dynamic response by using a multiscale DWT to locate instances of damage. Numerical examples of the method verified that single and double instances of damage could be located with satisfactory accuracy, but multiple damage and fast speed of the moving load reduced the accuracy of localization. Gökdağ [87] combined the CWT and DWT methods to analyze the measured displacement response from a cracked beam subjected to a moving load for damage location. The DWT was used to extract a suitable approximation function from the data, which was extended from the measured data of a damaged beam to reduce boundary distortion. The CWT was used to define a sensitive DI that was utilized to locate damage via the difference between the CWT coefficients of the original data and the approximation function. The DI was computed with the extended f(t) and the $A_{J}(t)$ extracted from f(t) as follows [87]:

(21) $$I_{Ⅰ}=W_{f}(s,b)-W_{A}(s,b),$$

where f(t) is the time displacement of a point or the time-dependent strain; $A_{J}(t)$ is a suitable approximation function extracted from f(t); $W_{f}$ and $W_{A}$ refer to the CWT coefficients of f(t) and $A_{J}(t)$, respectively.

(22) $$I_{Ⅱ}=W_{f}(s,b)-W_{f}^{u}(s,b),\;I_{Ⅲ}=W_{f}(s,b),$$

where the superscript u indicates the CWT coefficients of the time-dependent response of the intact beam. The indices above are computed so that they can be compared with the DI $I_{I}$. $I_{I}$ extracts reference data from the damaged structural response via the DWT, whereas $I_{II}$ employs that from the CWT. Numerical applications of the proposed method indicated that strain data was more sensitive to damage than displacement data and that the centrifuge and Coriolis forces among the moving mass-related force terms could improve the damage sensitivity of both displacement and strain data.

### 3.3. WPT-Based Methods

#### 3.3.1. Definition of the WPT

The wavelet package transform (WPT) can be defined as [88,89]:

(23) $$\{\begin{array}{l} u_{2n}^{\left(j\right)}(t)=\sqrt{2}\underset{k}{\sum }h(k)u_{n}^{\left(j\right)}(2t-k)\\ u_{2n+1}^{\left(j\right)}(t)=\sqrt{2}\underset{k}{\sum }g(k)u_{n}^{\left(j\right)}(2t-k) \end{array};\;n,k\;=\;0,1,2,\cdots ,$$

where $u_{0}^{\left(0\right)}(t)=\varphi (t)$ denotes the scaling function, $u_{1}^{\left(0\right)}(t)=\psi (t)$ refers to the wavelet function, j, k, n are the decomposition level, the translation parameter, and the modulation parameter, respectively. $H=\;{\left\{h(k)\right\}}_{k\in Z}$ and $G={\left\{g(k)\right\}}_{k\in Z}$ are corresponding function sets that imply the low-pass and high-pass filters respectively; h(k) and g(k) are quadrature mirror filters. The recursive relations between the jth- and the j+1th-level components of the WPT are given in the equations [90]:

(24) $$\begin{array}{c} f_{j}^{i}(t)=f_{j+1}^{2i-1}(t)+f_{j+2}^{2i}(t),\\ f_{j+1}^{2i-1}(t)=\;Hf_{j}^{i}(t),\\ f_{j+1}^{2i}(t)=Gf_{j}^{i}(t). \end{array}$$

After being decomposed j times, the original signal f(t) can be expressed by the sum of the component signals as [90]:

(25) $$f(t)=\overset{2^{j}}{\underset{i=1}{\sum }}f_{j}^{i}(t).$$

Unlike the WT, which merely covers the lower-frequency part, the WPT provides more even and exhaustive decomposition along the entire frequency scope because each remaining detail signal is decomposed again in a similar manner to the decomposition of the approximation signal component.

#### 3.3.2. WPT for Damage Detection Subjected to Moving Loads

Under the heading of crack identification using the CWT and DWT, WPT methods have also been employed in damage detection subjected to moving loads. Many researchers have conducted damage identification, location, and quantification using wavelet packet decomposition [91,92,93,94,95]. Some have used the WPT for damage detection in beam-like bridges subjected to an impact load or cyclic combined loading [96,97]. Few have applied the WPT in beams subjected to a moving load to locate damage. Zhang et al. [98] utilized the WPT for crack identification based on the deflection response at a single point of a beam subjected to moving loads. The WPT splits a signal not only on the low-frequency content but also on the high-frequency content, for which signals are transformed by multiresolution analysis (MRA) known as the DWT. The signal component of the WPT branch reflects the various corresponding frequencies in the time domain. Use of the WPT can result in the detection of the singularity point caused by the damage in the operational deflection time history by which the abnormality in the WPT coefficients represents the crack location. In this method, the energy of the abnormal signal of the wavelet packet branch is related to a DI, by which damage severity can be estimated. The proposed approach was validated as effective for crack location only when an appropriate wavelet was selected and used. Sun et al. [99] employed the local sample entropy of a wavelet packet frequency band to propose displacement response analysis for crack identification when a moving load passes the damage area. The wavelet packet was used to decompose the displacement time history data and reconstruct the appropriate frequency band coefficients by taking the standard frequency band energy. Then, the DI was calculated from the sample entropy value of each local interval, which was derived from the reconstructed signal. The results of the method showed that: (1) the index was able to identify multiple damage and to estimate the relative degree of damage in different locations; (2) the closer the measuring point was to the damage zone, the higher was the value of the index; and (3) the method could directly detect damage using the signals in the time domain with fewer measurement points and flexible positions.

To conclude, the WT serves as a time-frequency analysis technique that provides more detailed information regarding nonstationary signals than traditional Fourier analysis. The FT has limitations for signal processing in nonstationary processes. The FT can obtain the composition of the frequencies of an entire signal, but it has no knowledge of the time of appearance of the ingredients. The advantages of the WT technique are its ability to capture multiscale information and reflect characteristic signal changes in both time and frequency scales. Hence, wavelet analysis is very suitable for analyzing nonstationary signals, which in turn makes it feasible for the signal processing underlying damage detection. However, the signal processing method “Hilbert-Huang transform” (HHT), discussed in the next section, is suitable for both structural health monitoring (SHM) and damage detection.

## 4. HHT-Processed Dynamic Responses to Moving Loads for Damage Identification

The HHT [100] has attracted considerable attention as a recently developed signal processing technique. As a novel time-frequency analysis method, the HHT can analyze nonlinear and nonstationary time series of measured signals [101,102]. The technique has been effectively employed to process structural dynamic responses for damage assessment [103,104,105]. Quek et al. [106] adopted an HHT-based approach to detect structural anomalies in beams and plates; Li et al. [107] applied the HHT to analyze vibrational signals and for fault diagnosis of roller bearings. Recently, the HHT has been applied to the detection of structural damage caused by moving loads, typically for locating and quantifying damage [110,111,112,113,114,115,116,117].

### 4.1. Definition of the HHT

The HHT includes two joint technical components: empirical mode decomposition (EMD) [100,108] and the Hilbert transform. EMD can decompose a measured response signal x(t) into “intrinsic mode functions” (IMFs). The procedure of EMD is to construct upper and lower envelopes of the signal by spline fitting and then to compute the mean of both envelopes. Then, a sifting process is performed by subtracting the signal from the mean. After that, the sifting process is repeated until the resulting signal becomes a monocomponent signal, referred to as an IMF. The original signal is then subtracted from the IMF signal, and the iterative sifting process is applied to the remaining signal to obtain another IMF signal. The process recurs to obtain n IMFs, i.e.,:

(26) $$x(t)=\sum_{j=1}^{n}c_{j}(t){+r}_{n}(t),$$

in which $c_{j}(t)\left(j=1,2,\ldots ,n\right)$ denote the IMFs of the measured signal x(t) and $r_{n}(t)$ is a residue containing either the mean trend of the original signal or a constant. This process is called the EMD method [100].

After acquiring IMFs by the EMD method, the Hilbert transform is applied on each IMF component [109]:

(27) $$H\left[c_{i}(t)\right]=\frac{1}{\pi }\int_{-\infty }^{+\infty }\frac{c_{i}(\tau )}{t-\tau }d\tau .$$

The Hilbert-Huang spectrum H(ω,t) is designated as:

(28) H(ω,t)=Re∑i=1nai(t)exp(j∫ωi(t)dt).

### 4.2. IMF of Dynamic Responses to Moving Loads

Damage identification using IMFs arising from EMD to tackle structural dynamic responses to moving loads has been investigated by Meredith, Bradley, and Aied et al. [110,111,112]. Methods for damage detection of civil structures subjected to moving loads based on EMD are as follows. Meredith et al. [110] employed the IMFs of the acceleration response of a beam subjected to a moving load to identify multiple damage locations. The IMFs of the measured response were converted from the measured signal by EMD. The distinctive spike of the IMF indicated discontinuity caused by a sudden loss of stiffness in the structure. Bradley et al. [111] applied a moving average filter followed by a high-pass filter to the acceleration signal to preprocess the signal, yielding pure signals. The new signal-based model-free and adaptive method utilized the nonlinear characteristics of the response and the transient properties of the load to locate damage without prior knowledge regarding the structure. The method was able to detect a crack of 10% depth of the beam, using a signal generated at the moving load velocity of 10 m/s. The effectiveness of the method in detecting more than one damaged section was analyzed in a variety of scenarios, including a range of bridge lengths, various velocities of the moving load, and different noise levels. Nevertheless, the results of the method could be enhanced by using multiple sensors to improve the accuracy of damage prediction. Aied et al. [112] proposed an ensemble empirical mode decomposition (EEMD) [113] to produce refined IMFs from the acceleration responses of a bridge subjected to moving loads. The EEMD featured stronger ability than the EMD to separate high-frequency components from other frequency components of nonlinear signals. High frequencies are commonly related to sudden stiffness changes in structures. The use of refined IMFs to identify sudden stiffness changes in bridges was verified by accurate identification of stiffness changes in a bridge despite the small size of affected regions, relatively poor profiles, high vehicle speeds, and significant noise.

### 4.3. HHT-Processed Dynamic Responses to Moving Loads

Despite the prevalence of HHT utilization in damage detection, few studies have focused on the use of this technique to identify damage in structures subjected to moving loads. Representative investigations are briefly examined here. Liu et al. [114] used the HHT method to analyze the displacement responses of a beam under the effect of a moving load for damage detection. The HHT first used the EMD to decompose the displacement signal data into IMFs. The IMFs were converted by the Hilbert transform to extract analytic signals, from which instantaneous frequency (IF) curves were obtained. An abrupt change in the IF curve delineated the damage location. Numerical simulations of the HHT showed that damage could be located with relative accuracy. Roveri and Carcaterra [115] employed the HHT to analyze the dynamic deflection responses measured at a single point of a beam experiencing a travelling load for damage location and quantification, with no baseline information required. The deflection response was decomposed into a series of IMFs through EMD, and the IMFs were transformed by the Hilbert transform to extract the analytic signals. To address a high-frequency wave, the Hilbert transform of a harmonic signal $w_{h}(t)$ with a piecewise continuous amplitude produced an approximate result as follows [115]:

(29) $$H[w_{h}(t)]\approx {c\Omega w}_{1}(t)[e^{j\omega_{\Omega }(t-t_{d}+T)}\sin c(2\Omega (t-t_{d}+T))],$$

where $c=\frac{\omega_{1}-\omega_{v}}{\omega_{1}}$, $f_{\Pi }=\frac{2V}{L-L_{1}}$, $\Omega =\pi (f_{\Pi }-f_{1})$, $\omega_{\Omega }=\pi (f_{1}+f_{\Pi })$, $\omega_{v}=V\sqrt[{4}]{\rho A/EI}\sqrt{\omega_{1}}$, $t_{d}=L+L_{1}/2V$, $T=L-L_{1}/2V$, t=L/V and $\omega_{v}<\omega_{1}<\omega_{\Omega }<\omega_{\Pi }$; $\omega_{1}$ denotes the angular frequency of the first mode; L denotes the length of the beam; $L_{1}$ refers to the crack location from the left edge of the beam; V denotes the velocity of the moving load; $f_{1}$ denotes the fundamental frequency. In particular, the speed of the moving load should satisfy a definite range for the applicability of the proposed identification technique: $\frac{L-L_{1}}{2}f_{1}<V<\frac{4}{3}Lf_{1}$. Then, damage can be located by directly observing the highest peak of the first instantaneous frequency that presents a sharp crest corresponding to the damaged section. Theoretical and numerical results showed good accuracy of the method in locating the damaged section. However, the identification was insensitive to damage extent, crack location, or ambient noise when it was limited by the velocity range of the moving load. Chouiyakh et al. [116] proposed methodological approaches based on the HHT and the differential quadrature method (DQM) to analyze the vibrational deflection responses of multiply cracked Timoshenko beams subjected to moving masses for multi-crack identification. The DQM-based method was used to deal with the effects of a piecewise domain and the coupling effects resulting from multiple cracks and the moving mass. A time-DQM method was developed to conduct multimodal analysis and present a numerical crack identification procedure. The multi-crack identification procedure based on the HHT of eigenmodes and time deflection was demonstrated to be effective and accurate. Li and Hao [117] used the HHT to analyze the dynamic responses of measured relative displacement and acceleration for damage detection of a composite bridge model subjected to moving loads. Numerical studies and experimental verifications of the methods were conducted to validate that both relative displacement and acceleration measurements could identify the location and the instant of damage occurrence in a composite bridge subjected to moving loads. Moreover, relative displacement was manifested as a better response quantity for damage identification in the composite bridge.

In conclusion, the HHT is a suitable tool for processing nonstationary wave propagation signals collected in damage detection studies. Good representation of localized events in both the frequency and energy of any measured transient signal is provided by the HHT. Thus, the HHT is a simple signal processing tool that can provide reasonably good results. It includes two main parts: the first part is the EMD proposed by Huang [100] and the second part is the Hilbert spectrum analysis. It relies mainly on the EMD, which permits decomposition of the acquired signal into a set of basic functions called IMFs. IMFs are processed through the Hilbert transform to obtain the Hilbert-Huang spectrum that elicits time-frequency features of the analyzed data. Thus, characteristic changes in the structural system can be tracked using the Hilbert-Huang spectrum. Nevertheless, application of the HHT for damage detection in bridges subjected to moving loads has not been fully saturated. The HHT suffers some implementation problems, such as insensitivity to the speed of the moving load. Hence, researchers have explored other methods to interrogate damage in bridges subjected to moving loads.

## 5. Dynamic Responses-Driven HID

The aforementioned FT-, WT- and HHT-based identification of damage in bridges subjected to moving loads have the common feature that the dynamic response is directly processed to characterize damage. Rather than direct processing of dynamic responses to moving loads, the dynamic responses or their derivatives can be indirectly processed to interrogate damage, invoking a procedure of heuristic interrogation of damage (HID) of damage in bridges subjected to moving loads. The basic components of HID include dynamic responses from the actual bridge and a simulated bridge model involving damage parameters, residual of dynamic responses or their derivatives between actual and simulated bridges, objective functions underpinned by the residual, and an optimization algorithm such as genetic algorithm (GA) [118,119,120], particle swarm optimization (PSO) [121,122,123], or neural networks [124,125]. The philosophy of the HID is that the dynamic responses are input to the HID procedure to drive the optimization algorithm, and the optimization algorithm progressively minimizes the objective function by adjusting the damage parameters to interrogate damage heuristically [137,138,139,140]. Typical dynamic responses-driven HID procedures for damage diagnosis in bridges subjected to moving loads can be categorized in terms of the optimization algorithm into two groups, GA-based HID and PSO-based HID.

In structural damage detection for HID, the theoretical foundation is formulation of the residuals for dynamic characteristics between an intact structure and a cracked structure. Dynamic characteristics can be obtained from the dynamic responses of the structure. By acquiring dynamic responses from the intact and cracked structures respectively, residuals can be represented as the difference between the dynamic responses of the two types. The residuals reflect the deviation of the structure with damage, suitable for formulating the objective function for damage interrogation driven by dynamic responses.

### 5.1. Type-I Residual Relying on Displacement Responses

Simulated and experimental displacement responses have been defined as [126]: $Y=\left(y-y^{0}\right)/\sqrt{\left(\sum_{i=0}^{N-1}y^{0}{\left(t_{i}\right)}^{2}\right)/N}$; $Y^{x}=\left(y^{x}-y^{0}\right)/\sqrt{\left(\sum_{i=0}^{N-1}y^{0}{\left(t_{i}\right)}^{2}\right)/N}$, respectively. With the discrepancy $Y^{x}-Y$ between the displacement responses, the residual γ can be expressed by [126]:

(30) $$\gamma =f(Y^{x}-Y),$$

where f is a filter that would be used to optimize the residual; y and $y^{x}$ denote the theoretical or simulated measurement and experimental measurement, respectively; $y^{0}$ represents the undamaged state of the bridge specimen; $y^{0}\left(t_{i}\right)$ is a discrete function in the time domain at N sampling points. On the basis of γ, the objective function can be formulated as [126]:

(31) $$F=\frac{1}{2}{\left|\gamma \right|}^{2}=\frac{1}{2}\frac{1}{N_{i}}\sum_{i=1}^{N_{i}}\gamma_{i}^{2},$$

where $N_{i}$ is the size of the residual vector γ. To improve sensitivity while approaching the optimum and enhance the convergence speed of optimization, an algorithm and a small value ε are introduced: $F^{l}=\log(F+\varepsilon )$.

On the above basis, Rus and Lee [126] utilized a GA algorithm to minimize the objective function in Equation (31) for damage identification and quantification in bridge decks subjected to moving loads. The GA algorithm was utilized to improve the sensibility of the probability of detection (POD), as well as the computational efficiency and competence. The proposed estimation of the POD was a function of uncertainty conditions of the noise and system, the location and extent of the damage, and the objective function previously presented. POD estimation and a spatial damage model accurately representing the stiffened degradation area were used to present an optimal filtering for damage detection. That study can supply guidelines and criteria for bridge design and contribute to the reliability and robustness of model-based SHM. However, more practical influences such as damping and resonance effects should be taken into consideration.

Alternatively, the residual was defined as the relative difference between the measured and computed displacement responses [127]:

(32) $$\gamma {=}^{i}Y{-}^{i}U(q),$$

and the objective function or fitness function was expressed as [127]:

(33) $$\mathrm{minimize}(F),\;F=\sum_{i=1}^{N}{\left[\gamma \right]}^{2}=\sum_{i=1}^{N}{\left[{}^{i}Y{-}^{i}U(q)\right]}^{2},$$

where ${}^{1}Y,\;\ldots ,{}^{N}Y$ denote the measured displacement data; ${}^{i}U:\;R^{s}\to R\left(i=1,\;\ldots ,\;N\right)$ is the function that satisfies $U:\;\Omega \subset R^{s}\to \Gamma \subset R^{N}$; $U(q)={\left[{}^{1}U^{2}U\;\ldots {\;}^{N}U\right]}^{T},\;q\in \Omega$; Ω, s, N, Γ denote the vector space of identification variables, the number of identification variables, the vector space of dynamic displacement responses, and the number of measured displacement data points used, respectively; ${}^{1}U,\;\ldots ,{\;}^{N}U$ represent dynamic displacement responses calculated by using the dynamic analysis combining the finite element method (FEM) with μGA from an arbitrary *q*.

Lee et al. [127] used μGA and the FEM to analyze measured dynamic displacement responses for identifying the arbitrary stiffness distribution in damaged reinforced concrete bridges subjected to moving loads. The dynamic displacement responses with noise effects were measured from a 3D solid finite element model subjected to a moving load with various velocities instead of real reinforced concrete slab bridges. On the basis of the measured responses, a modified bivariate Gaussian distribution function was utilized to analyze the distribution of the stiffness reduction. The μGA adopted in the study was used to reduce the computational time for the calculations using FEM solvers and simplified complex problems including model and data errors. Using the combined finite element analysis and μGA, the distribution of deteriorated stiffness for damage interrogation could finally be determined by investigating the unknown parameters q. Numerical results of the method showed that the proposed procedure was feasible and practical for inspecting the complex distribution of deteriorated stiffness but not sensitive to high movement velocities. Similar studies were conducted by Lee et al. [128] and Park et al. [129] to identify damage in bridges, with the results showing that the damage could be detected with high accuracy.

### 5.2. Type-II Residual Relying on Displacement Responses

The residual was regarded as the difference between the dynamic displacement response of the damaged beam and that of the mathematical model of the intact beam [130,131]:

(34) $$\gamma =y(z_{n},t)-\bar{y}(z_{n},t).$$

The objective function based on the residual could then be defined as [130]:

(35) $$F(x)=\sum_{n=1}^{N_{\mathrm{mp}}}\int_{0}^{T}\frac{\left|y(z_{n},t)-\bar{y}(z_{n},t)\right|}{\max(\left|\bar{y}(z_{n},t)\right|)}dt,$$

where $N_{mp}$, $z_{n}$, T denote the number of measurement points, the location of the *n*th measurement point, and the total time for the vehicle to move across the beam, respectively; $\bar{y}$, y refer to the reference displacements measured from the damaged beam and the corresponding displacements computed by the mathematical model of the beam; x denotes the vector containing crack location and size parameters, i.e., $x=\left\{{\bar{h}}_{1},{\bar{h}}_{2},\ldots ,{\bar{h}}_{N_{c}},{\bar{z}}_{1},{\bar{z}}_{2},\ldots ,{\bar{z}}_{N_{c}}\right\}$, ${\bar{h}}_{i}=\frac{h_{i}}{h}$, ${\bar{z}}_{i}=\frac{z_{i}}{L}$, where $N_{c}$, $h_{i}$, $z_{i}$, h, L denote the number of cracks, the depth of the ith crack, the distance of the ith crack from the left edge, the entire depth of the beam, and the entire length of the beam, respectively.

Gökdağ [130,131] utilized PSO to analyze dynamic displacement responses for damage localization and quantification in beam-type structures subjected to a moving vehicle. Based on Equation (35), the optimization problem could be expressed as [130]:

(36) $$\begin{array}{l} \min F(x)\\ subject\;to:\\ {\bar{z}}_{i}-{\bar{z}}_{i+1}<0,\;i=1,2,\ldots ,N_{c}-1\\ 0<{\bar{z}}_{j}<1,0\leq {\bar{h}}_{j}<1,j=1,2,\ldots ,N_{c} \end{array}\}.$$

To determine crack locations and depths, PSO with linearly increasing inertial weight was employed to solve Equation (36). The results of numerical simulations showed that cracks with the depth ratio of 0.1 could be detected with an error level up to 3%.

Based on the foregoing study, Gökdağ [132] used the multiresolution property of the DWT to enhance the damage detection capacity of PSO for the identification of small cracks. To enhance the performance of the PSO method, the measured reference displacements (RDs) in Equation (34) were added in a sequence of random numbers to simulate the effect of experimental noise arising from instrumentation, environmental conditions, numerical errors, and so forth [132]:

(37) $$\bar{y}{\left(z,t\right)}_{\mathrm{noisy}}=\bar{y}(z,t)+N_{p}\cdot G\cdot \sigma ,$$

where $\bar{y}(z,t)$ is the calculated RD, which is free of noise, z is the point on the damaged beam. $N_{p}$, G, σ denote the noise percentage, Gaussian distribution with zero mean and unit standard deviation, and the standard deviation of $\bar{y}(z,t)$, respectively. To alleviate noise, the multiresolution property of the DWT was exploited. Then, the objective function in Equation (35) could be revised as [132]:

(38) $$F_{2}(x)=\sum_{n=1}^{N_{\mathrm{mp}}}\int_{0}^{T}\frac{\left|y(z_{n},t)-{\bar{y}}_{M}(z_{n},t)\right|}{\max(\left|{\bar{y}}_{M}(z_{n},t)\right|)}dt,$$

where ${\bar{y}}_{M}$ denotes the approximation function of $\bar{y}$ at the Mth decomposition level in the DWT. On this basis, the modified method could detect small cracks with a depth ratio of 0.1 despite 5% noise interference; moreover, it could identify small damage in bridges with much more accuracy than the traditional CWT coefficients method [68,83].

On the basis of Equations (35) and (36), Gökdağ [133] investigated three versions of PSO, namely linearly decreased inertial weight (LDIW) PSO [134,135,136], linearly increased inertial weight (LIIW) PSO [137], and constriction factor (CF) PSO [138], for crack identification in beams subjected to moving loads. To improve the PSO convergence speed, LDIW initializes the inertial weight with a higher value to promote exploration in the early stages of optimization and decreases it linearly to a smaller value to eliminate oscillatory behaviors in later stages. In contrast, LIIW increases the inertial weight from a lower value to a higher one iteratively. In both implementations, a small inertial weight facilitates the exploration of new space and a large inertial weight provides more opportunities to stabilize the algorithm. The CF induces particles to converge on local optima and prevents swarm explosion, which is a common problem in traditional PSO algorithms. In comparisons of the performance of the three PSO versions, all the algorithms produced accurate results for damage detection subjected to moving loads. However, CF and LIIW had similar best values, both of which were better than that obtained from LDIW. Likewise, the convergence speeds and robustness of LIIW and CF were similar and superior to that of LDIW. LIIW and CF were nearly the same for most of the cases considered but CF was slightly better than LIIW from the point of view of robustness. The three PSO algorithms were then compared to the CWT coefficients [68,81,83,87] for crack identification in bridges subjected to moving loads. It was observed that PSO could identify cracks with a depth ratio equal to 0.15 despite 3% noise interference. The method could also detect cracks in beams under higher moving load velocities for which the CWT coefficients failed to locate damage.

### 5.3. Type-III Residual Relying on Acceleration Responses of the Bridge

The residual in Equation (32) was used by Noh and Lee [139] for processing the acceleration and displacement of a concrete slab bridge subjected to unknown moving loads. The displacement data in Equations (32) and (33) was replaced by the acceleration data. Both the acceleration and displacement responses of the bridge were analyzed for comparison. From the comparisons, acceleration-response-based detection was more practical than displacement- or velocity- response-based detection because acceleration data could be obtained directly from normal accelerometers. Unlike the procedure in reference [127], a hybrid GA was used instead of the μGA to optimize the residual of Equation (32). The hybrid GA was formulated by the incorporation of a GA with a conventional gradient-based optimization technique in light of the advantage of the former in enhancing the searching capability and that of the latter in improving convergence performance. The hybrid GA, based on the finite element analysis, could characterize the change of structural properties and loading condition in bridges. The capability of the hybrid GA was verified by numerical examples, showing that it could precisely predict the characteristics of damage at a 5% noise level despite modeling and measurement errors.

### 5.4. Type-IV Residual Relying on Acceleration Responses of Moving Loads

The residual has been defined as the discrepancy between the acceleration responses of a vehicle moving on a bridge under damaged and intact states [140]:

(39) $$\gamma ={\ddot{q}}_{2}^{mu}-{\ddot{q}}_{2}^{s},$$

Then the objective function could be set as [140]:

(40) $$F={\left\|{\ddot{q}}_{2}^{mu}-{\ddot{q}}_{2}^{s}\right\|}_{2},$$

where ${\ddot{q}}_{2}^{mu}$, ${\ddot{q}}_{2}^{s}$ denote the acceleration response of a vehicle moving on the damaged and intact bridge, respectively.

Li and Au [140] proposed a multistage multipass strategy based on the modal strain energy and a GA to locate damage in a continuous bridge from the vertical acceleration responses of a vehicle moving on the bridge. By obtaining the vehicle’s acceleration responses, the frequencies of both intact and damaged bridges could be extracted from the acceleration responses using an empirical mode decomposition method. On the basis of the extracted frequencies, a damage indicator was proposed by employing a modal strain energy method to evaluate the locations of damage. The estimated locations were then globally optimized by GA techniques for accurate damage identification. Using the function Equation (40), the GA could roughly locate damage based on the acceleration responses from multiple passes of the vehicle. Numerical results demonstrated that the combined method could locate singular damage with acceptable noise influences. The measurement noise had significantly less influence on damage detection than the roughness of the bridge. There is still a need for intensive study of this strategy with multiple locations of damage.

PSO and GAs have much in common, such as random initialization of a population, evaluation of a system using an adaptive value, and performance of a particular random search according to a fitness value. However, PSO does not include genetic operations such as crossover and mutation; rather, it uses its own speed to decide the search [141]. From the study of Sandesh and Shankar [142], PSO proved to be fast, whereas GA performed worst in speed and accuracy.

It is observed that HID methods have numerous advantages compared to traditional calculations. They do not require the establishment of an exact mathematical or logical model of the problem itself but deal directly with the results of input data. HID methods are more applicable for solving problems that traditional artificial intelligence technology finds difficult to deal with effectively or simply cannot handle. Among HID methods, GAs can be used in various combinations. However, the efficiency of GAs is lower than that of other traditional optimization methods, and such methods tend to converge prematurely. PSO, alternatively, does not exhibit the problems encountered by GAs. Research has shown that PSO is a type of optimization algorithm with considerable potential, which can converge more quickly and obtain superior results. Nevertheless, PSO also has some disadvantages: its performance in certain applications is not good, and the encoding of the network and the choice of the genetic operators can cause some difficulty. However, when there is only numerical data to be used, the neural network comes in handy. To summarize, HID is a robust area of technology that has more capability than traditional computing. In future, more and more diverse HID methods may be used in SHM and damage detection in structures subjected to moving loads.

## 6. Recommended Research Directions

This study provides a concise survey of the state of the art of damage identification in bridges by processing dynamic responses to moving loads. Four method types for processing dynamic responses are covered, with three direct methods: the FT, WT, HHT, and one indirect method: HID. The compared functions of the four methods are shown in Table 1. In particular, their features and uses for identifying damage in bridges subjected to moving loads are thoroughly investigated, with emphasis on the feasibility, efficacy, and influential factors of each method in dealing with dynamic responses to moving loads for damage characterization. It should be noted that identification of damage in bridges subjected to moving loads is a complex scientific and technological issue, for which resolution of some problems is still pending. To advance this research area, potential research directions are recommended as follows:

(1) Proper dynamic responses for damage detection in bridges should be produced by a moving load in an appropriate velocity interval [115,143]. Within that velocity interval, damage responses to moving loads feature superior robustness to ambient noise. Therefore, choice of the proper speed within the velocity interval is critical to damage detection in bridges subjected to moving loads.
(2) Nonlinear factors in structural dynamics have considerable effects on structural dynamic characteristics [144]. Thus, the nonlinear softening behavior of reinforced concrete bridges should be considered in damage detection of such bridges. With this intention, nonlinear approaches should be developed for damage detection in reinforced concrete bridges subjected to moving loads.
(3) The environmental components involved in dynamic responses can obscure damage characterization. It is necessary to eliminate environmental components before processing dynamic responses to moving loads.
(4) Sensor networks attached in bridges and moving loads produce multiple types of measured data. Multi-sensor data fusion algorithms [145,146,147,148] are promising for the processing of the multiple dynamic response types of bridges subjected to moving loads.
(5) A more versatile algorithm should be developed for considering several vehicles’ moving on bridges at different speeds.

## Figures and Tables

**Table 1:** Functions of four methods for detecting damage.

Methods	FT	WT	HHT	HID
**Functions**	Decompose a function of time (a signal) into the frequencies	Transform a time signal into a time-frequency domain, characterize local features of the signal	Decompose a signal into IMF along with a trend, obtain instantaneous frequency data, applicable to analyze nonstationary and nonlinear signal	Process the residual for generating high-quality solutions to optimization
